# The structural model of mother-infant bonding in the first pregnancy based on the mother’s attachment style and parenting style: the mediating role of mentalization and shame

**DOI:** 10.1186/s40359-023-01436-4

**Published:** 2023-11-16

**Authors:** Reza Yazdanimehr, Abdolaziz Aflakseir, Mehdireza Sarafraz, Mohammadreza Taghavi

**Affiliations:** Department of Clinical Psychology, Faculty of Educational Sciences and Psychology, University of Shiraz, Shiraz, Iran

**Keywords:** Pregnancy, Parenting, Mentalization, Shame, Bonding

## Abstract

**Introduction:**

This study aimed to evaluate the relationship between the mother’s attachment style and parenting style and mother-infant bonding in the first pregnancy considering the mediating role of mentalization and shame.

**Methods:**

This was a descriptive-correlational study. The sample population included the women who had gone through their first pregnancy and were referred to the health centers in Neyshabur, Iran in 2022. In total, 330 women were selected by convenience sampling. To collect data, we used a demographic questionnaire, the Attachment Style Questionnaire, the Parenting Style Questionnaire, the Mother-Infant Bonding Scale, the Reflective Functioning Questionnaire, and the Guilt and Shame Proneness Scale. Data analysis was conducted using descriptive indices, correlation coefficients, and pass analysis.

**Results:**

Overall, the findings showed that the studied model adequately fits the data. Further, the obtained results confirmed the mediating role of mentalization and shame in the relationship between the mother’s attachment style and parenting style and mother-infant bonding. A significant correlation was also observed between attachment styles, parenting styles, and mother-infant bonding (*p* < 0.01).

**Conclusion:**

Mother-infant bonding is correlated with the mother’s attachment style/parenting style, shame, and mentalization. Thus, we can help vulnerable mothers by improving the quality of psychological care before the first pregnancy or during pregnancy.

## Introduction

Pregnancy is a complex and important process in a woman’s life and may be accompanied by psychological and physiological changes. Childbirth is considered a significant transition into the mother-infant relationship [1] Taking care of the infant and fulfilling his needs can be largely influenced by the mother’s early experience of being taken care of, as well as the quality of her attachment style [2]. Therefore, special attention should be paid to the behavioral and psychological aspects of the mother-infant relationship.

Mother-infant bonding is an important relational pattern with a central role in the infant’s survival and growth. Mother-infant bonding refers to the emotional bonding between the mother and infant, which begins to develop before childbirth [3]. This process is a critical factor regarding the mother’s adjustment to the changes brought about by pregnancy and accepting the responsibilities of motherhood. There are no accurate statistics regarding the problems associated with mother-infant bonding. Only one study reports that 3% of mothers do not enjoy their relationship with their infants [4]. The relational pattern between the mother and infant can be influenced by the mother’s experience of her original family. What a mother internalizes from her relationship with her parents is an internal working model deeply ingrained in the behavioral and motivational systems governing a child’s attachment to their mother. These internal working models serve as the foundational rules through which an individual comes to comprehend their identity in relation to others and learns to regulate their emotions. Consequently, these models lead individuals to replicate the same behavioral patterns they experienced as children when interacting with their own offspring [5]. Attachment styles are one of the variables that are associated with the individual’s early experiences; attachment styles may develop to be either secure or insecure (avoidant or anxious). Previous studies showed a relationship between secure and insecure attachment styles and the quality of mother-infant bonding. For instance, Nordal et al. report a relationship between the quality of mother-infant bonding and the quality of the mother’s attachment; accordingly, mothers with insecure attachment styles (anxious/avoidant) often have more relational problems with their infants [6]. Moreover, Moghadam Hosseini studied mothers with 1-month-old infants, observing that the mother’s attachment style is correlated with how attached the mother is to the infant [7]. Mothers with secure attachments exhibit a heightened sense of importance and attentiveness to others, thereby enhancing their capacity for caregiving. Indeed, possessing such an internal working model in adulthood, especially during significant life transitions like becoming a mother, equips them with the necessary preparedness to nurture a newborn [8].

In addition to attachment, the parenting procedures perceived by parents are significant predictors of our important relationships as adults. The study undertaken by Baumrind proposes a remarkable approach in this regard [9]. He believes that parenting styles have two dimensions: control and acceptance. Combining these two components, Baumrind developed three main parenting styles, including the authoritative parenting style, the authoritarian parenting style, and the permissive parenting style. Several studies confirm that the individual’s experience of her parents’ parenting style has an impact on her life and relationships as an adult. According to Allen et al., individuals with authoritarian parents often face challenges in their interpersonal relationships [10].

When the individual first becomes a parent, mother-infant bonding exposes the mother to different challenges. To better understand the process, we need to properly analyze different variables that can affect the pattern of the mother-infant relationship. The importance of the developmental context lies in the fact that changes in one’s conditions can lead to changes in the person’s interactions and subsequently his relationships. The mother’s ability to recognize the infant’s mental states and distinguish them from her mental states is a factor that plays a central role in the mother-infant relationship and shows the impact of the mother’s developmental experiences. This ability is commonly referred to as “mentalization” [11]. It becomes evident that the levels of acceptance and responsiveness in authoritative parents facilitate infant care and responsiveness to their needs. In contrast, mothers with authoritarian parenting styles exhibit weaker verbal and non-verbal communication with their infants and are less responsive to the unique needs of their babies [12]. The mothers’ internal image form about parenting in relation to their own parents significantly influences their ability to establish a connection and care for their own child [13]. During the first year after childbirth, a mother must harmonize herself both mentally and physically with the infant’s developmental changes to sustain a supportive interaction with her baby. An assertive parenting style enables a mother to better synchronize herself with her infant, whereas an authoritarian parenting pattern often results in a lack of understanding of the baby’s needs and desires, leading to attunement [14]. What underscores the significance of parenthood in the interaction between mother and infant is its innate capacity to facilitate the creation of a nurturing environment. Optimal parenting empowers the mother to assume a distinct role as a caregiver in the infant’s psychological world. In such circumstances, the mother’s dedication to the infant, the quality of care provided during this period, and the emotional investment in the infant all contribute to fostering a strong bond between the mother and the newborn. Social cues such as talking, soothing, and playing further enhance this connection [15].

The present mental state of mothers, considering their caregiving experiences within the context of attachment and childhood parenting, provides a valuable yet incomplete perspective on their bond with the infant. While the influence of attachment and caregiving experiences continues to shape mothers throughout their development, it alone does not elucidate all the pathways to emotional bonding with others. The pattern of attachment transmission and its effects on the mother-infant bond do not conform to a linear model; instead, a multi-causal model should be considered. Recent findings over the past decade have shifted the focus from the concept of ‘inheritance’ in attachment development to the influence of interactive factors. This encompasses simultaneity, reciprocity, synchrony, and optimizing emotional exchanges during initial mother-infant interactions [16].

Paying full attention to the emotions experienced in a given situation and within a group is crucial, as it helps new mothers empathize with their unprocessed and accumulated emotions as well as those of their infants. When new mothers witness their infants going through emotional distress, challenging situations, or discomfort, processing their own experiences becomes vital to maintaining emotional attunement with the infant. This enables them to recognize the unique and individual needs of the infant, distinct from the time when the infant was inside them as a separate individual, and value those needs empathetically [17]. Pregnancy and subsequent motherhood create an opportunity for women to demonstrate their mentalization abilities. One of the fundamental components in the mother-infant relational process is the presence of the child in the mother’s mind. This presence assists the growing infant in achieving a shared mental and physical connection. Mentalization, resulting from reflective functioning and emotional regulation, illuminates this process. Both the mother and the infant require support to create emotional experiences together. Consequently, the infant becomes aware of their own presence in the mother’s mind, perceived through subtle nuances in the emotional meanings conveyed by the mother’s responses to sounds, gestures, and the baby’s facial expressions. The infant needs to understand that their mother possesses a mentality, and they, too, have a mind that they can utilize throughout their life, nurturing its growth and employing it to comprehend themselves and others in their interactions [18]. Mentalization in parents pertains to their capacity to understand their mental states and the ability to bear the child’s mental states in mind [19]. According to the literature, mothers with a greater capacity for mentalization often bond better with their infants [20]. The mother’s mentalization plays a pivotal role in her taking care of the infant. As long as the infant’s relationship with the mother is solely nonverbal and the parent interprets the child’s inner world through observing behavioral and emotional indications, the mother’s reflected empathetic responses promote the infant’s understanding that his emotions are acceptable and manageable [21].

Due to the physical changes it involves, the first pregnancy defines a new role for a woman, as well as new care responsibilities (e.g., breastfeeding an infant), which bring about wide-ranging emotions. Shame is an emotion that can be associated with the physical changes caused by pregnancy and the newly defined roles (e.g., the experience of feeding an infant) for women [22]. Shame is defined as the internalization of feeling of guilt toward oneself, particularly when the individual perceives herself as a failure in others’ eyes. According to Taylor, perceiving oneself as a “bad mother” while facing the experience of feeding an infant is a universal phenomenon [23]. Other studies show that a mother may feel ashamed when she fails to fulfill her tasks of taking care of or feeding her infant [24]. The experience of shame disrupts the attunement and synchrony in the mother-infant relationship. Shame-triggered incongruities lead to thoughts related to a flawed self-concept in the mother, impacting the experience of ‘self’ within the relationship. When this connection is severed or when one is left alone, the chronic sense of fractured selfhood gives rise to deep feelings of isolation, hopelessness, and worthlessness. In such instances, the mother feels a sense of worthlessness in her interactions with the infant and others around her, losing her capacity for empathy. She perceives a lack of solidarity and believes that others are not empathetic, thus relinquishing her own capacity for empathy [25]. Therefore, individuals must become aware of their central role as parents or caretakers throughout this complex process. As the first year of life is critical in the development of attachment styles, the quality of the mother-infant relationship is the best context to create a secure attachment in the infant. Previous studies have mainly focused on a specific level of attachment, giving the child the most important role in this regard and neglecting other interpersonal levels. Investigating different levels of attachment processing and the influential factors can result in the integration of the available studies. The current research aims to contribute to the integration of existing studies by examining various levels of attachment processing and the related influential variables. Given the inadequate data on parental motivations, the development of internal working models in the parent as inspired by the infant, and its impact on the mother-infant relationship, clarifying the correlation between the variables associated with the mother-infant relationship quality can be of clinical importance for screening processes. Further, such studies lay the groundwork for therapeutic and preventive interventions during and after pregnancy. Therefore, the current study seeks to investigate whether mentalization and shame mediate the relationship between attachment style and maternal parenting style in the context of mother-infant bonding.

## Methods

### Study design and participants

This descriptive-correlational study was conducted based on structural equation modeling (SEM). The sample population consisted of all the women who had gone through their first pregnancy and were referred to the health centers in Neyshabur, Iran in 2022. The inclusion criteria of the study were as follows: 1) women who went through their first pregnancy; 2) being in the postpartum period of 2–12 months; 3) the age range of 18–45 years; 4) minimum education level of middle school; 5) no history of mental disorders and chronic physical conditions and 6) not receiving any medication therapy or psychotherapy within at least the past 6 months. The exclusion criteria were as follows: 1) unwillingness to complete the questionnaires and 2) providing incomplete information. The sample size was determined based on Kline’s suggestion [26], which proposes a minimum sample size of 300 in SEM. Considering a 10% drop out, the sample size was set to 330 participants.

### Data collection

After obtaining an introduction letter from Shiraz University and making the necessary arrangements for the study, four health centers were selected conveniently from the health centers in Neyshabur city. The researcher referred to the selected centers and obtained a list of the women who had undergone their first pregnancy. Following that, participants were invited to the study through announcements sent out in the centers by an expert midwife. After the participants were selected, they were justified about the study goals, how to complete the questionnaires, issues around informed consent (e.g., the individual consent of each subject to participate in the study), being allowed to withdraw from the study in case of unwillingness to continue participation, and confidentiality terms. After ensuring that the participants would not become physically/mentally tired, they were asked to sign the consent form and complete the questionnaires in a quiet environment without any disturbance. In total, 330 participants aged 18–36 years were evaluated in the present study. In terms of age 129 participants (39.1%) were < 24, 159 participants (48.2%) were 25–29, and 42 participants (12.7%) were > 30 years. The mean and standard deviation of their age were respectively 25.67 and 3.39 years. In terms of education, 102 participants (30.9%) had a high school diploma, 173 participants (52.4%) had a bachelor’s degree, 50 participants (15.2%) had a master’s degree, and 5 participants (1.5%) had a Ph.D. In terms of occupation, 181 participants (54.8%) were employed, and 149 participants (45.2%) were unemployed.

### Measures

#### Adult attachment style questionnaire

It is an 18-item scale developed by Collins and Read in 1990 based on the theoretical principles of the attachment theory. This scale evaluates the respondent’s relational skills and intimate relationships based on a Likert scale. The score of each item ranges from 1 (Completely Disagree) to 5 (Completely Agree). The questionnaire has three subscales, namely secure attachment, anxious attachment, and avoidant attachment. Items 1, 6, 8, 12, 13, and 17 are in the secure attachment subscale, items 3, 4, 9, 10, 11, and 15 are in the anxious attachment subscale, and items 2, 5, 7, 14, 16, and 18 are in the avoidant attachment subscale. Based on the obtained score, the respondent’s attachment style is classified as either secure, anxious, or avoidant. Notably, items 5, 8, 16, 17, and 18 are reversely scored within the range of 0–4. The validity of this scale has been confirmed for the Iranian population. In their study, Pakdaman and Khanjani reported Cronbach’s alpha to be 0.81, 0.78, and 0.85 for the secure attachment, avoidant attachment, and anxious attachment subscales, respectively. In the current research, the Cronbach’s alpha values for the subscales of secure attachment, avoidant attachment, and anxious attachment were obtained as 0.84, 0.75, and 0.78, respectively [27].

#### Postpartum mother-infant bonding scale

This 25-item questionnaire was developed by Brockington in 2006. It consists of four components, namely mother-infant pathological bonding, rejection and anger, infant-focused anxiety, and risk of child abuse. The items are scored based on a Likert scale ranging from zero (Never) to 5 (Always). The minimum and maximum scores are respectively 0 and 125, with the higher scores showing problematic mother-infant bonding. The cutoff point of the entire scale is 38. The validity and reliability of this questionnaire have been confirmed for the Iranian population. Accordingly, Cronbach’s alpha is reported to be 0.52, 0.67, 0.70, 0.74, and 0.87 for the components of pathological bonding, rejection and anger, infant-focused anxiety, risk of child abuse, and the total scale, respectively. In the current study, the Cronbach’s alpha coefficient for the overall scale was 0.85, and for the subscales of pathological bonding, rejection and anger, infant-focused anxiety, risk of child abuse, the coefficients were obtained as 0.62, 0.71, 0.68, and 0.76, respectively [28].

#### Parenting style questionnaire

This 30-item questionnaire was developed in 1991 based on Baumrind’s theory of permissive, authoritative, and authoritarian behavioral patterns. The permissive parenting style is measured by items 1, 6, 10, 13, 14, 17, 19, 21, 24, and 28, while the authoritative parenting style is assessed by items 2, 3, 7, 9, 12, 16, 18, 25, 26, and 29, and the authoritarian parenting style is evaluated by items 4, 5, 8, 11, 15, 20, 22, 23, 27, and 30. The items are scored based on a five-point Likert scale. The questionnaire has been normalized and validated for the Iranian population. Also, Cronbach’s alpha is respectively estimated at 0.78, 0.81, and 0.88 for the permissive parenting style, authoritarian parenting style, and authoritative parenting style, confirming the reliability of the scale. In the current research, the Cronbach’s alpha coefficients for the subscales of authoritative parenting, authoritarian parenting, and permissive parenting were obtained as 0.81, 0.78, and 0.74, respectively [29].

#### Reflective functioning scale

It is a self-report tool to measure the respondent’s mentalization ability. Fonagy et al. developed this tool in 2016. In a factor analysis, they discovered and reported the two factors of certainty and uncertainty regarding the mental state of the individual and others. This questionnaire consists of 14 items, which are scored based on a seven-point Likert scale. The scale has been normalized for the Iranian population. Also, Cronbach’s alpha confirms the reliability of the scale for the certainty factor (0.88), the uncertainty factor (0.66), and the total scale (0.85). In the current study, the Cronbach’s alpha coefficient for the overall scale was 0.82, for the factor of certainty it was 0.84, and for the factor of uncertainty, it was 0.71 [30].

#### Guilt and shame proneness scale

This 16-item scale was developed by Cohn et al. in 2011 to measure two dimensions: guilt and shame. To use this scale, participants present narratives of the everyday situations they might face and explain their reactions to these situations. Following that, the participants are asked to imagine themselves in the same situations and rate the probability of reacting to the situations based on a five-point scale ranging from 1 (Rarely) to 5 (Very Often). The questionnaire has two guilt subscales: negative evaluations (items 1, 9, 14, and 16) and repair action tendencies (items 2, 5, 11, and 15). There are also two shame subscales, including negative self-evaluations (items 3, 6, 10, and 13) and withdrawal action tendencies (items 4, 7, 8, and 12). The psychometric validity of this scale has been evaluated in the Iranian population. Further, the analysis of the internal consistency reliability of the questionnaire in the study by Hashemi showed Cronbach’s alpha coefficients of guilt, shame, and the total scale to be 0.82, 0.79, and 0.86, respectively. In the current research, the Cronbach’s alpha coefficients for the overall scale, guilt, and shame were obtained as 0.86, 0.77, and 0.83, respectively [31].

### Statistical analysis

Data analysis was conducted using descriptive indices, correlation coefficients, and pass analysis. Data collection tools are above below.

## Results

### Descriptive indices of variables

Tables 1 and 2 shows the descriptive factors and correlation matrix. The mean secure attachment style was 18.87 ± 5.11, the mean anxious attachment style was 14.84 ± 6.35, and the mean avoidant attachment style was 12.79 ± 3.18. The mean values of other variables are presented in Table 1. According to the information in Table 2, mother-infant bonding was significantly correlated with secure (− 0.79), anxious (0.55), and avoidant attachment styles (0.43) and authoritarian (0.72), permissive (− 0.42), and authoritative parenting styles (0.70) (*p* < 0.01). More correlations between the variables are shown in Table 2. To test the assumptions of path analysis for assessing the normality of variables, skewness and kurtosis were used. In the hypothetical model, the observed variables had a range of absolute skewness from 0.74 to 0.96 and kurtosis ranging from 0.09 to 1.61. The values obtained for skewness and kurtosis of the variables indicate the achievement of the normality assumption for each individual variable. Furthermore, to investigate the assumption of linearity, in addition to correlation matrices, statistical tests like Variance Inflation Factor (VIF) and Tolerance Index were employed. Given that the correlation coefficients were not higher than 0.85, and none of the VIF values were less than 10, and none of the variance inflation factors exceeded 10, it can be concluded that the assumption of non-collinearity is met. Additionally, in the current study, to identify univariate outliers for the observed variables, frequency tables and box plots were used. To identify multivariate outliers, Mahalanobis distances were calculated for each participant, and as a result, none of the participants were excluded from the analysis.

**Table 1:** Descriptive indices of variables

Variable	M (SD)	Sk	Ku
1. Secure attachment style	17.87 (5.11)	−0.53	− 1.16
2. Anxious attachment style	14.84 (6.35)	0.96	−0.42
3.Avoidant attachment style	79.12 (3.18)	0.67	0.1
4. Authoritative parenting style	40.32 (6.74)	−0.67	−0.09
5. Permissive parenting style	26.05 (4.87)	−0.43	0.57
6. Authoritarian parenting style	26.65 (6.42)	0.07	−0.88
7. Mentalization	57.58 (10.70)	−0.45	−1.07
8. Shame	18.88 (6.73)	0.53	−0.96
9. Mother-infant bonding	41.90 (14.84)	0.58	−0.79

**Table 2:** Correlation matrix of variables

Variable	1	2	3	4	5	6	7	8	9
1. Secure attachment style	1								
2. Anxious attachment style	−0.65**	1							
3.Avoidant attachment style	−0.37**	−.13*	1						
4. Authoritative parenting style	0.70**	−.44**	−0.38**	1					
5. Permissive parenting style	0.38**	−0.26**	−0.25**	0.27**	1				
6. Authoritarian parenting style	−0.67**	0.45**	0.36**	−0.77**	−0.25**	1			
7. Mentalization	0.79**	−0.53**	−0.39**	0.74**	0.40**	−0.64**	1		
8. Shame	−0.78**	0.56**	0.41**	−0.63**	0.45**	0.63**	−0.72**	1	
9. Mother-infant bonding	−0.79**	0.54**	0.43**	−0.71**	−0.41**	0.69**	−0.77**	0.82**	1

*P < 0.05 **P < 0.01

### Fit indices of structural model

Table 3 shows the fit indices of the final model. According to Table 3, the fit indices of the structural model indicate an appropriate fit of the model. For instance, the Comparative Fit Index (CFI), Goodness of Fit Index (GFI), and Incremental Fit Index (IFI) in the current model were 0.99, 0.99, and 0.98, respectively, all of which exceed the 0.90 benchmark for these indices, indicating a good fit of the model. Finally, the Root Mean Square Error of Approximation (RMSEA), which is one of the most important fit indices, was 0.07, which is less than the 0.08 benchmark for model fit. In summary, the fit indices indicate a good fit of the model to the data.

**Table 3:** Fit indices of structural model

Fit indices	Acceptable range	Suggested model	Obtained model
χ2	–	7.08	16.52
χ2/df	< 3	7.08	2.75
CFI	> 90%	0.99	0.99
IFI	> 90%	0.99	0.99
GFI	> 90%	0.99	0.98
RMSEA	< 0.08	0.13	0.07
SRMR	< 0.08	0.007	0.01

### Path coefficients of direct effects of variables

Accordingly, the final pattern of the research model was well-fitting. Table 4 shows the unstandardized coefficients, standardized coefficients, the standard error, the *t* statistic, and the level of significance to determine the significance of the path coefficients between the variables. Accordingly, all the variables the direct paths of which to the dependent variable has a higher or lower *t* value than ±1.96 have significant effects on the dependent variable. According to Table 4, it can be observed that the direct paths from secure attachment style variables (*T* = 0.13, *β* = 0.11) and avoidant attachment style to the mother-infant bonding variable are significant (*T* = 0.31, *β* = 0.08). However, the direct path from anxious attachment style variable to the mother-infant bonding variable is not significant (*T* = 0.70, *β* = 0.07). Furthermore, based on Tables 4, it can be seen that the direct paths from authoritative parenting style variables (*T* = 0.51, *β* = 0.12) and authoritarian parenting style to the mother-infant bonding variable are significant (*T* = 0.58, *β* = 0.11). However, the direct path from permissive parenting style variable to the mother-infant bonding variable is not significant (*T* = 0.12, *β* = 0.03).

**Table 4:** Path coefficients of direct effects of variables and significance of estimated parameters

Independent variable	Dependent variable	Unstandardized coefficient	Standardized coefficient	Standard error	t	P
Secure attachment style	Mother-infant bonding	−0.34	− 0.11	0.16	−2/13	0.03
Anxious attachment style	Mother-infant bonding	0.16	0.07	0.09	1.70	0.08
Avoidant attachment style	Mother-infant bonding	0.38	0.08	0.16	2.31	0.02
Authoritative parenting style	Mother-infant bonding	−0.26	−0.12	0.10	−2.51	0.01
Permissive parenting style	Mother-infant bonding	−0.10	−0.03	0.09	−1.12	0.26
Authoritarian parenting style	Mother-infant bonding	0.25	0.11	0.1	2.58	0.01
Mentalization	Mother-infant bonding	−0.19	−0.14	0.06	−2.90	0.004
Shame	Mother-infant bonding	0.87	0.39	0.10	8.70	0.001
Secure attachment style	Mentalization	0.89	0.42	0.11	7.72	0.001
Anxious attachment style	Mentalization	−0.15	−0.08	0.07	−1.94	0.05
Avoidant attachment style	Mentalization	−0.29	−0.08	0.13	−2.28	0.02
Authoritative parenting style	Mentalization	0.58	0.36	0.07	7.35	0.001
Permissive parenting style	Mentalization	0.23	0.10	0.07	3.30	0.001
Authoritarian parenting style	Mentalization	0.04	0.02	0.08	0.49	0.61
Secure attachment style	Shame	−0.51	−0.39	0.07	−6.65	0.001
Anxious attachment style	Shame	0.22	0.21	0.05	4.39	0.001
Avoidant attachment style	Shame	0.41	0.19	0.08	4.8	0.001
Authoritative parenting style	Shame	−0.06	−0.06	0.05	−1.12	0.26
Permissive parenting style	Shame	−0.22	−0.16	− 0.04	−4.73	0.001
Authoritarian parenting style	Shame	0.11	0.11	0.05	2.14	0.32

### Mediating effect of mentalization and shame variables

Table 5 shows the mediating effects of the mentalization and shame variables associated with the correlation of attachment styles and parenting styles with mother-infant bonding based on the bootstrap method with 2000 sampling processes at a 95% confidence interval. According to Table 5, it can be observed that the indirect effect of secure attachment style variables (*p* < 0.05, *b* = 0.174), anxious attachment style (*p* < 0.05, *b* = 0.03), and avoidant attachment style (*p* < 0.05, *b* = 0.058) on the mother-infant bonding variable through mentalization is significant. Similarly, the indirect effect of authoritative parenting style variables (*p* < 0.05, *b* = 0.11) and permissive parenting style (*p* < 0.05, *b* = 0.5) on the mother-infant bonding variable through mentalization is significant. However, the indirect effect of authoritarian parenting style variable on the mother-infant bonding variable through mentalization is not significant (*p* > 0.05, *b* = 0.008).

**Table 5:** Mediating effect of mentalization and shame variables in relationship of attachment styles and parenting styles with mother-infant bonding

Independent variable	Mediating variable	Dependent variable	Nonstandard coefficient	Upper limit	Lower limit	*P*
Secure attachment style	Mentalization Mentalization	Mother-infant bonding	−0.17	−0.32	− 0.05	0.007
Anxious attachment style	Mentalization	Mother-infant bonding	0.03	0.002	0.08	0.03
Avoidant attachment style	Mentalization	Mother-infant bonding	0.05	0.01	0.15	0.01
Authoritative parenting style	Mentalization	Mother-infant bonding	−0.11	−0.22	−0.03	0.006
Permissive parenting style	Mentalization	Mother-infant bonding	−0.04	−0.10	− 0.01	0.005
Authoritarian parenting style	Mentalization	Mother-infant bonding	−0.008	−0.05	0.02	0.46
Secure attachment style	Shame	Mother-infant bonding	−0.45	−0.67	− 0.27	0.001
Anxious attachment style	Shame	Mother-infant bonding	0.2	0.11	0.31	0.001
Avoidant attachment style	Shame	Mother-infant bonding	0.36	0.2	0.58	0.001
Authoritative parenting style	Shame	Mother-infant bonding	−0.05	−0.14	0.02	0.19
Permissive parenting style	Shame	Mother-infant bonding	−0.19	−0.30	− 0.10	0.001
Authoritarian parenting style	Shame	Mother-infant bonding	0.10	0.01	0.19	0.009

Furthermore, based on Tables 5, it can be seen that the indirect effect of secure attachment style variables (*p* < 0.05, *b* = 0.45), anxious attachment style (*p* < 0.05, *b* = 0.2), and avoidant attachment style (*p* < 0.05, *b* = 0.36) on the mother-infant bonding variable through shame is significant. Similarly, the indirect effect of permissive and authoritarian parenting style variables (*p* < 0.05, *b* = 0.1) on the mother-infant bonding variable through shame is significant. However, the indirect effect of authoritative parenting style variable on the mother-infant bonding variable through shame is not significant (*p* > 0.05, *b* = 0.05).

The results indicate that in the final model, compared to the proposed model, mentalization plays a mediating role in the relationship between secure attachment styles and authoritative and permissive parenting styles with mother-infant bonding. However, these results were not significant in relation to the authoritarian parenting style. Similarly, the results show that shame plays a mediating role in the relationship between attachment styles and permissive and authoritarian parenting styles with mother-infant bonding, but this mediation was not significant in relation to the authoritative parenting style.

**Fig. 1:**
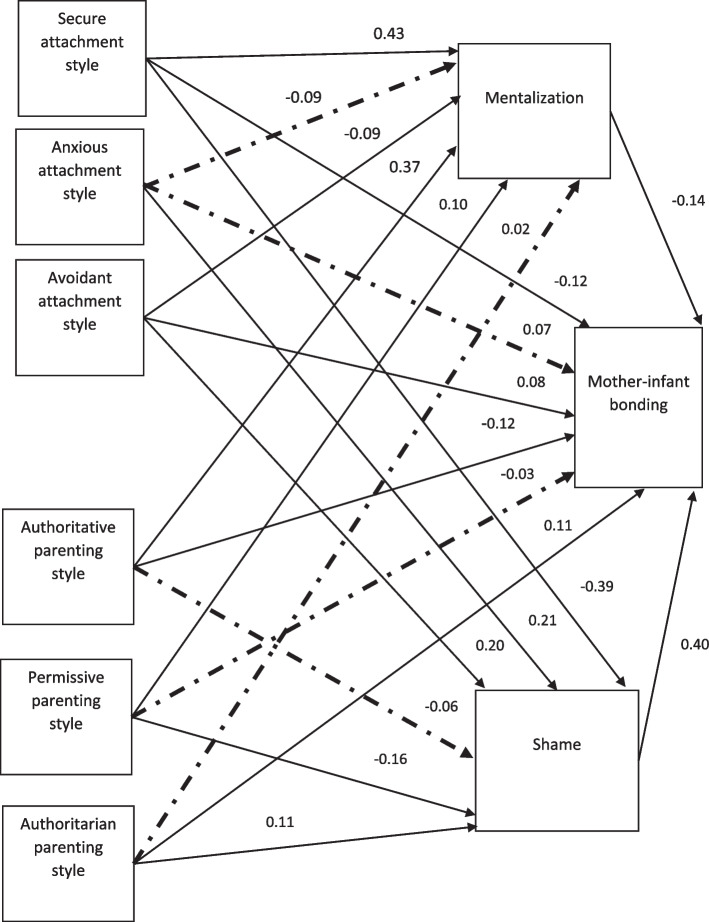
Conceptual research model and standardized path coefficients

Figure 1 depicts the conceptual research model and the standardized path coefficients of the variables. The significant paths are shown with solid lines, and the dotted lines represent the non-significant paths.

## Discussion

The results of the present study showed that maternal attachment styles are directly correlated with mother-infant bonding. This is in line with the studies by Nonnenmacher [32], Nordal [6], Nakano et al. [33], Wawrzkiewicz et al. [34], and Moghadam Hosseini [35]. These findings can be explained in the context of the attachment theory. Mothers with a secure attachment often pay more attention to and sympathize with others, which, in turn, improves caring for others. Using this internal working model in adulthood, especially in the critical stages involving change (e.g., becoming a mother), increases the individual’s capacity and preparedness to take care of an infant [18]. Previous studies show that mothers with an insecure attachment (anxious and avoidant) have a negative understanding of mother-infant bonding due to the unpleasant experience of their relationship with their mothers and being deprived of a very important care-related factor (i.e., a secure and responsive relationship) [8]. While experiencing motherhood for the first time, the mother’s mental representations of her parents in the past coincide with her self-image and image of her infant. Thus, the mother’s early childhood attachment experience is correlated with her mental representation of her infant, which may influence the quality of the mother-infant relationship [36].

The results of the present study also showed that mentalization plays a mediating role in the relationship between attachment styles and mother-infant bonding. This is in line with the studies by Grienenberger [37], Arnot [38], Ensink [39], and Dollberg [40]. The ability of parents to mentalize the infant’s psychological states serves as a tool to develop an attachment between the parent and the infant [41]. There is a reciprocal relationship between mentalization and attachment. In other words, a secure attachment bond facilitates the individual’s mentalization ability, whereas an insecure attachment decreases this ability. Further, mentalization abilities facilitate a secure attachment bond in both the parent and the child. Mentalization is manifested as the mother’s active, solemn effort to observe and identify the underlying mental states that lead to the infant’s behavior. These states provide the mother with the ability to verbally express herself and create a firm bond between the mental states and behaviors of the infant and herself within a “two-way” communication context. As effective as this ability is in improving mother-infant bonding, the lack of such capacity can equally disrupt maternal functioning while bonding with the infant [42].

Our findings showed that shame plays a mediating role in the relationship between secure attachment styles and mother-infant bonding. This is in line with the previous studies in this regard. For instance, Gross [43] and Akbag [44] reported that mothers with a secure attachment experience low levels of shame and adopt effective emotion regulation strategies if they experience feelings of shame. Mothers with a secure attachment generally have positive feelings toward themselves and others. They believe emotional distress is manageable, and this approach helps them be open to the possible experience of shame, consider it an adaptive emotion, and better cope with the feeling [45].

The obtained results showed that shame plays a mediating role in the relationship between the insecure attachment style (avoidant and anxious) and mother-infant bonding. This is in line with the study by Shaver and Mikulincer [46], which showed that mothers with an avoidant attachment style often face more challenges in bonding with their infants. In mothers with an insecure attachment (avoidant and anxious), shame can be characterized by increased worries about being seen or exposed to others, as well as a feeling of inadequacy for the new role as a mother. Mothers with an avoidant attachment avoid emotional stimuli. As a result, when shame increases due to these states, their avoidance and withdrawal intensify, thereby decreasing the quality of the mother-infant relationship [47]. Further, mothers with an anxious attachment style are constantly worried about their feelings toward others and face emotional ups and downs in their interpersonal relationships [48]. To these mothers, even the slightest challenge in taking care of an infant causes extreme worry, and they find it difficult to believe in themselves and their capabilities. Through shame, an insecure attachment bond prevents the development of optimal attunement between the mother and infant, and a satisfactory bond becomes unavailable.

The obtained results showed that mother-infant bonding is inversely correlated with the authoritarian parenting style, while it is directly and significantly correlated with the authoritative parenting style. This is in line with the studies by Shieh [14], Madden et al. [49], and Fraiberg [12], which showed that the mother’s caretaking and parenting experiences in the past are associated with the quality of bonding with the infant. To clarify, we should mention two components that play a central role in parenting; responsiveness and control. It seems that high levels of acceptance and responsiveness in authoritarian parents facilitate the care of the infant, as well as responding to his care needs. Mothers with an authoritarian parenting style tend to have more emotional capacity, which helps them promptly respond to the care needs of the infant and improve their relationship with him. In contrast, mothers with an authoritative parenting style stress control and obedience. It also seems that they have poor verbal and nonverbal communication with the infant and are less responsive to the unique needs of the infant [13]. Authoritative mothers show less emotional intimacy and are unable to acknowledge the specific needs of the infant. Moreover, these mothers are emotionally unavailable, which predisposes them to more problems in their relationships, including their relationship with the infant [50]. Our findings regarding the permissive parenting style were not significant. Although the study by Zelenski [51] showed that permissive parents often have a better relationship with their children, this correlation was not found significant in the present study. There may be other hidden factors involved (e.g., culture), which require further studies.

According to the results of the present study, mentalization plays a mediating role in the association of the authoritarian parenting style and the permissive parenting style with mother-infant bonding. Previous studies show that mentalization is correlated with positive parental behaviors (e.g., sensitivity), the improvement of the child’s emotional development, and the absence of hostility in the mother [37, 52, 53]. The authoritarian and permissive parenting styles influence mother-infant bonding through mentalization when mentalization creates an awareness of the mental states of oneself and others, recognizes the underlying causes of the behavior, and identifies the needs and intentions of oneself and others, thereby helping the mother understand and tolerate the infant’s distressful emotional experiences and provide optimal care and security to the infant. When the mother demonstrates a certain amount of mentalization, she can regulate her emotional state while interacting with the infant. In this case, mentalization helps the mother understand the infant’s behavior based on his mental state and improves the quality of her relationship with him [37]. Our findings regarding the authoritative parenting style were not significant, which implies that mentalization alone may not influence the relationship between these two variables. Further, these mothers may suffer from hidden vulnerabilities, which can act as intervening factors.

The results showed that shame plays a mediating role in the association of the permissive parenting style and the authoritative parenting style with mother-infant bonding. However, this association was not considered significant in terms of the authoritarian parenting style. The findings of this study showed that the direct correlation between the permissive parenting style and mother-infant bonding is not significant, while it becomes significant through shame as a mediating variable. This is inconsistent with the previous studies in this regard. The studies by Gilbert [54] and Gross [43] showed that shame is associated with the low quality of mother-infant bonding. This discrepancy can be attributed to the cultural component. Shame is experienced differently in various communities and cultures. Wurmser [55] refers to the experience of shame as protective shame, which prevents facing disgrace and is characterized by a humble, respectful attitude. It seems that in mothers with this parenting style, the experience of shame, along with a component of acceptance they may have, helps facilitate the mother-infant relationship. Given the lack of sufficient and reliable studies regarding the cultural component of shame in Iran’s society, further studies must be conducted to closely examine this component.

Mothers with an authoritative parenting style tend to look stern and cold when feeling ashamed, which disrupts mother-infant bonding as facial expressions and the messages the mother facially conveys to the infant are extremely important [56]. If looked closely at, shame contains the mother’s negative experiences of her relationships while she was being taken care of; if her understanding of authoritative care is accompanied by shame, the quality of her relationship with the infant decreases [57]. A mother with feelings of shame is like an empty mirror that cannot reflect anything; in an authoritative mother with a limited emotional capacity, this diminishes her ability to feel related to the infant [58]. The mediating role of shame in the relationship between the authoritarian parenting style and mother-infant bonding was not significant in the present study. This may be because shame is a complex emotion encompassing several other emotions or because there are other intervening factors involved that have not been identified yet. The results of this study are promising in the sense that in the Iranian society, given its unique cultural conditions and nuances, it sheds new light on the importance of maternal mental health for research pathways in this field. Additionally, by understanding psychological factors such as a mother’s early experiences of caregiving and the quality of her relationship with her parents, based on components related to the quality of mother-infant bonding, it provides a foundation for designing proactive educational programs for new mothers or therapeutic interventions.

Although the results of this study showed a correlation between attachment styles, parenting styles, and mother-infant bonding, and mentalization and shame play a mediating role in this correlation, the findings should be generalized with caution given the limited studies conducted in this regard in Iran. For more accurate conclusions, further studies are required. One of the limitations of the present study was sampling from only one city, which should be taken into account considering the cultural diversity of Iran so that the subject would be evaluated within a wider context. Further, the restrictions associated with the Covid-19 pandemic and health protocol obligations led to difficulties in the sample collection. Questionnaires were our main tools in this study; given the limitations of these tools, it is suggested that, if possible, interviews be used in further studies to obtain more accurate and in-depth data regarding the quality of the mother-infant relationship. Furthermore, since this study was conducted as a university project and due to constraints in terms of time, budget, and research execution conditions, the possibility of extending the duration of this study for longer-term investigations, additional comparisons, and further research was not available.

It is also suggested that further studies compare mother-infant bonding in the first pregnancy with subsequent pregnancies or compare mothers who have twins with those who have singletons. Also, mothers who have children of different genders can be studied to obtain more accurate and reliable results regarding the influential factors in mother-infant bonding.

## Conclusion

This study aimed to evaluate the association of the mother’s attachment style and parenting style with mother-infant bonding in the first pregnancy considering the mediating role of mentalization and shame. According to the results, mentalization and shame play a mediating role in the relationship of attachment styles and parenting styles with mother-infant bonding. Mother-infant bonding is one of the most fundamental relational experiences of every individual at the beginning of life. The outcomes of this relationship can persist throughout one’s life. What occurs in the context of attachment can be passed through generations. Thus, by psychologically preparing mothers for pregnancy and postpartum care, their mental health can be facilitated, which, in turn, improves the mental health of many generations to come.

## Data Availability

The datasets are available from the corresponding author on reasonable request.
